# 916. Increased efficiency and impact of implementing ILUM insight within an antimicrobial stewardship program (ASP) at an academic medical center

**DOI:** 10.1093/ofid/ofac492.761

**Published:** 2022-12-15

**Authors:** Ryan K Shields, Rachel V Marini, Sunish Shah, Bonnie A Falcione, Brian A Potoski, Leanna Liu, Eli S Goshorn, Lloyd Clarke, Alex Viehman, Christiane Hadi, Eun Jeong Kwak, Palash Samanta, Tina Khadem, J Ryan Bariola, Caley Yakemowicz, Courtney Simonick, Riaan Erwee, Erin K McCreary, Rima Abdel-Massih, Minh-Hong Nguyen

**Affiliations:** University of Pittsburgh, Pittsburgh, Pennsylvania; UPMC, Pittsburgh, Pennsylvania; Antibiotic Management Program, UPMC Presbyterian Hospital, Pittsburgh, PA, Pittsburgh, Pennsylvania; UPMC, Pittsburgh, Pennsylvania; UPMC, Pittsburgh, Pennsylvania; UPMC, Pittsburgh, Pennsylvania; UPMC, Pittsburgh, Pennsylvania; Antibiotic Management Program, UPMC Presbyterian Hospital, Pittsburgh, PA, Pittsburgh, Pennsylvania; University of Pittsburgh, Pittsburgh, Pennsylvania; UPMC, Pittsburgh, Pennsylvania; University of Pittsburgh Medical Center, Pittsburgh, Pennsylvania; UPMC, Pittsburgh, Pennsylvania; UPMC, Pittsburgh, Pennsylvania; UPMC, Pittsburgh, Pennsylvania; Infectious Diseases Connect, pittsburgh, Pennsylvania; Infectious Diseases Connect, pittsburgh, Pennsylvania; Infectious Disease Connect, Pittsburgh, Pennsylvania; UPMC, Pittsburgh, Pennsylvania; UPMC, Pittsburgh, Pennsylvania; University of Pittsburgh, Pittsburgh, Pennsylvania

## Abstract

**Background:**

An ASP is mandated for all hospitals and requires extensive resources with multidisciplinary collaboration. We measured the impact of implementing real-time decision support software (ILUM Insight) within our ASP.

**Methods:**

Our ASP has relied on prior authorization since 2002 and focused audit and feedback since 2015. In August 2021 we implemented to bring actionable data to front-line stewards. ILUM provides real-time notifications, organizes communications, and tracks patient-and provider-level data. We hypothesized that ILUM would increase the efficiency of ASP workflow and result in decreased antimicrobial utilization. We compared data 6 months before (8/20 – 1/21) and after (8/21 – 1/22) implementation. There were no significant staffing changes during either period.

**Figure:**
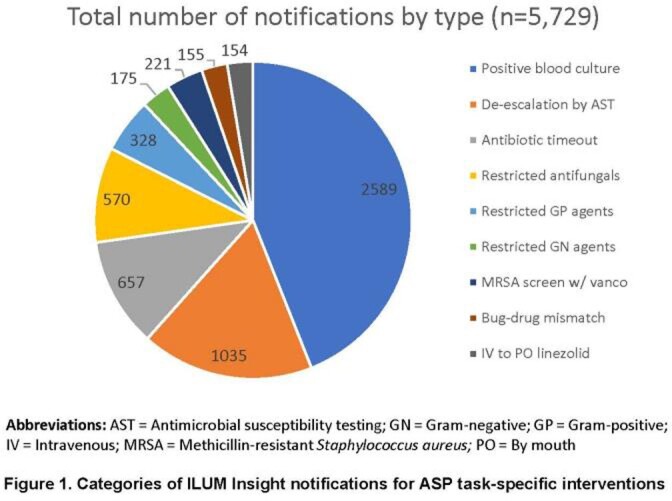

**Results:**

Existing notifications within ILUM were tailored to local practices, including alerts with intervention for positive blood cultures, antibiotic de-escalation, and bug-drug mismatches. New notifications were built for restricted antimicrobials, antibiotic timeouts, and MRSA screening. ASP pharmacists and physicians received training in July and November, respectively. A breakdown of all notifications received during the post-implementation period is provided in **Fig 1.** With increased ILUM usage, the number of interventions made by our ASP increased while missed opportunities decreased (**Fig 2.**). During the same time period, ASP communications rose from 205 to 1200 per month. Comparing pre- and post-implementation periods, antimicrobial days of therapy (DOT) per 1,000 patient days (PD) decreased by 14.5% from a median of 969 to 846 per month (**Fig 3;***P*=0.002). Antimicrobial expenditures were decreased by a median 21% per month during the post-intervention period compared to baseline. Among patients prescribed antimicrobials during an index admission, 30-day re-admissions decreased from 330 to 262 and re-admissions associated with re-ordering of antimicrobials decreased from 235 to 182 (**Fig 4**).

**Figure:**
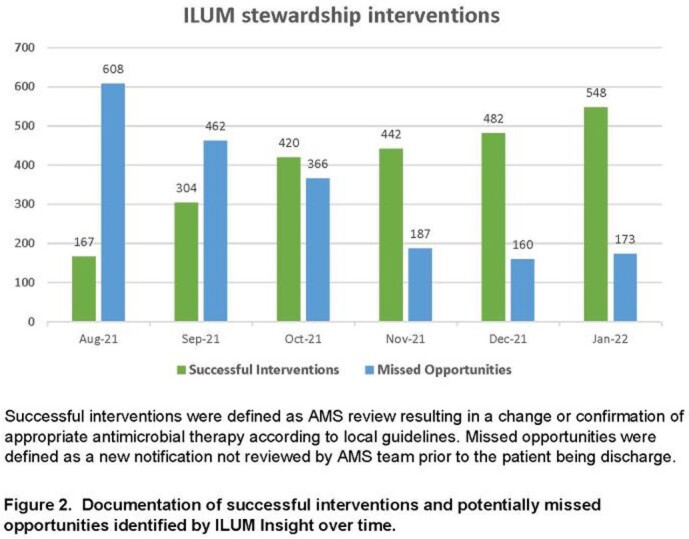

**Figure:**
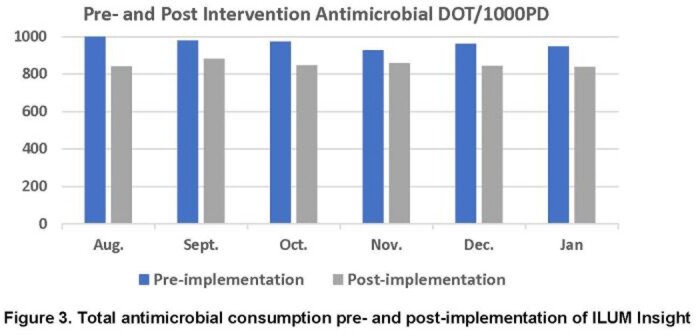

**Figure:**
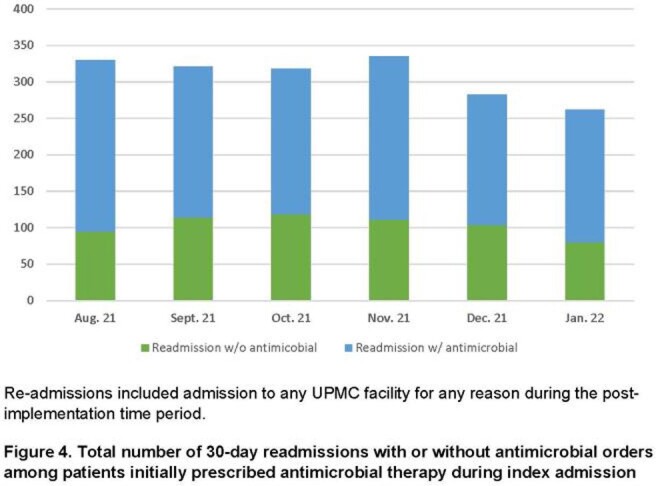

**Conclusion:**

Custom-designed, task-specific software improves the efficiency of daily ASP workflow and significantly decreased antimicrobial utilization without the need for additional ASP team members.

**Disclosures:**

**Ryan K. Shields, PharmD, MS**, Infectious Disease Connect: Advisor/Consultant|Merck: Advisor/Consultant|Merck: Grant/Research Support|Roche: Grant/Research Support **J Ryan Bariola, MD**, Infectious Disease Connect: Salary support|Merck: Grant/Research Support **Caley Yakemowicz, n/a**, Infectious Disease Connect: Employee **Courtney Simonick, n/a**, Infectious Disease Connect: Stocks/Bonds **Riaan Erwee, na**, Infectious Disease Connect: Employee **Erin K. McCreary, PharmD**, Infectious Disease Connect: Advisor/Consultant **Rima Abdel-Massih, MD**, Infectious Disease Connect: Co founder and Chief Medical Officer|Infectious Disease Connect: Ownership Interest.

